# Optimism measured pre-operatively is associated with reduced pain intensity and physical symptom reporting after coronary artery bypass graft surgery

**DOI:** 10.1016/j.jpsychores.2014.07.018

**Published:** 2014-10

**Authors:** Amy Ronaldson, Lydia Poole, Tara Kidd, Elizabeth Leigh, Marjan Jahangiri, Andrew Steptoe

**Affiliations:** a Department of Epidemiology and Public Health, University College London, 1-19 Torrington Place, London WC1E 6BT, United Kingdom; b Department of Cardiac Surgery, St. George's Hospital, University of London, Blackshaw Road, London SW17 0QT, United Kingdom

**Keywords:** Coronary artery bypass graft surgery, Optimism, Pain, Pessimism, Physical symptoms

## Abstract

**Objective:**

Optimism is thought to be associated with long-term favourable outcomes for patients undergoing coronary artery bypass graft (CABG) surgery. Our objective was to examine the association between optimism and post-operative pain and physical symptoms in CABG patients.

**Methods:**

We assessed optimism pre-operatively in 197 adults undergoing CABG surgery, and then followed them up 6–8 weeks after the procedure to measure affective pain, pain intensity, and physical symptom reporting directly pertaining to CABG surgery.

**Results:**

Greater optimism measured pre-operatively was significantly associated with lower pain intensity (β = − 0.150, CI = − 0.196 to − 0.004, *p* = .042) and fewer physical symptoms following surgery (β = − 0.287, CI = − 0.537 to − 0.036, *p* = .025), but not with affective pain, after controlling for demographic, clinical and behavioural covariates, including negative affectivity.

**Conclusions:**

Optimism is a modest, yet significant, predictor of pain intensity and physical symptom reporting after CABG surgery. Having positive expectations may promote better recovery.

## Highlights

• Optimism predicts better recovery after coronary artery bypass graft (CABG) surgery.
• We found that optimism was associated with lower pain intensity after CABG surgery.
• We found that optimism predicted fewer physical symptoms after the CABG procedure.
• Having positive expectations may promote better recovery following CABG surgery.

## Introduction

Optimism is a psychological trait characterised by positive expectations about future outcomes. It is a significant predictor of physical health [1] and is associated with enhanced physical recovery in a number of conditions and procedures such as traumatic brain injury, lung cancer, breast cancer, and bone marrow transplant [2]. The protective effects of optimism also extend to both pain and physical symptom reporting, and negative associations between optimism and pain have been reported in a number of chronic illnesses [3], [4], [5], [6]. Post-operative pain reporting is also lower among patients higher in optimism and this association has been demonstrated in patients who have undergone breast cancer surgery [7], and knee surgery [8], [9]. However, optimism was not found to predict pain in a large sample of patients undergoing elective surgery [10] and in some cases pessimism is found to be the more robust predictor of post-operative pain [11], [12].

Coronary artery bypass graft (CABG) surgery is a major procedure in terms of physical severity and patients typically experience pain and discomfort for up to 6 months after the operation [13]. A number of studies have examined the role of optimism in recovery following CABG surgery. Optimism has been shown to predict lower rates of rehospitalisation following CABG surgery [14]. Patients higher in optimism have also been found to have a faster rate of physical recovery and a higher quality of life at both 6 months [15] and 8 months after surgery [16]. The association between optimism and pain has been examined previously in CABG patients. It was found that those higher in optimism post-operatively experienced less pain in the weeks following the procedure [12]. However, optimism was measured 2–3 days after surgery, so optimism was not assessed prospectively.

Therefore in the current study, we planned to extend the literature in two main ways. Firstly, optimism was measured approximately four weeks prior to surgery, thereby avoiding the confounding effects of the procedure, and providing a more accurate baseline measure of dispositional optimism. Secondly, in addition to pain, we also measured physical symptoms using a patient-based measure specifically designed to assess coronary revascularisation outcomes. This measure allowed us to explore associations between optimism, pain, and elements of recovery specific to CABG surgery which has not previously been tested in the literature. We hypothesised that patients with higher levels of optimism would have less pain and fewer physical symptoms specific to CABG surgery 4–6 weeks post-operatively, controlling for several clinical factors and negative affectivity — a trait thought to confound optimism effects [17].

## Method

### Participants

The data we used in this study were collected as part of the Adjustment and Recovery after Cardiac Surgery (ARCS) Study. We carried out baseline assessments when patients attended the hospital for their pre-surgical clinic appointment on average 27.2 days before their surgery. Follow-up assessments took place on average 62.1 days after surgery and were carried out using postal questionnaires. Our inclusion criteria allowed only patients who were undergoing elective CABG surgery or CABG plus valve replacement to participate. CABG surgery included both on-pump and off-pump surgical procedures. Participants had to be able to complete the questionnaires in English, and be 18 years or older. All procedures were carried out with the written consent of the participants. Ethical approval was obtained from the National Research Ethics Service.

Participants were 265 prospective CABG patients who were recruited from a pre-surgery assessment clinic at St. George's Hospital, London. Follow-up data were collected 4–6 weeks after surgery from 215 patients; 2 patients had died, 13 had formally withdrawn from the study, and 35 did not respond to the follow-up questionnaires. Data on the three outcome measures were available for 201–215 patients. In order to carry out analyses of different outcomes on the same sample, we limited the analyses to 197 patients with complete data on all outcomes and covariates. There were no differences between patients who did and did not provide follow-up data in terms of age, gender distribution, ethnicity, smoking status, cardiological factors, total optimism levels, comorbidity or negative affectivity. But patients who were included in the analyses had lower body mass index (BMI) on average (28.3 versus 30.8, *p* < 0.001), and were less likely to have a history of diabetes (20.8% versus 36.9%, *p* = 0.013).

### Measures

#### Predictor: optimism

Optimism was measured at baseline using the revised Life Orientation Test (LOT-R), a 10-item self-report questionnaire that evaluates generalised expectations of positive and negative outcomes [18]. Participants were asked to indicate the extent of their agreement with each item (e.g. ‘In uncertain times, I usually expect the best’) from 0 (*strongly disagree*) to 4 (*strongly agree*). Six items (three reverse-scored) were used to derive the total optimism score, so ratings can potentially range from 0 to 24, with higher scores indicating higher levels of optimism. The remaining four questions on the LOT-R are filler items. Cronbach's alpha for the LOT-R for this sample was 0.68.

In sensitivity analyses, we tested separate optimism and pessimism subscales by summing the three items pertaining to optimism and three items pertaining to pessimism respectively. Ratings for each subscale can range from 0 to 12. Cronbach's alpha for the optimism subscale was 0.64 and 0.66 for the pessimism subscale.

#### Outcomes: affective pain, pain intensity, and physical symptom reporting

Two components of the McGill Pain Questionnaire — Short Form (MPQ-SF) [19] were used to assess affective pain and pain intensity in this study. The MPQ-SF was developed as a brief version of the standard MPQ and is suitable for use in post-surgical patients. The first component of the MPQ-SF that we used measures affective pain and is comprised of a list of 4 affective (e.g. fearful) descriptor words for which respondents are asked to rate their current experience of that particular type of pain from 0 (*none*) to 3 (*severe*). Responses are summed, ranging from 0 to 12, with higher scores indicating higher levels of pain. Cronbach's alpha for the affective pain subscale was 0.80.

The second component of the MPQ-SF that we used measures current pain intensity using a visual analogue scale. Using an adapted version of this scale, patients were asked at the follow-up assessment to rate their present pain intensity on a numerical rating scale ranging from 0 (*no pain*) to 10 (*worst possible pain*).

Physical symptoms were assessed at follow-up using the 11-item post-surgery physical symptom subscale from the Coronary Revascularisation Outcomes Questionnaire (CROQ) [20] designed for CABG surgery patients. This scale asks patients to rate the extent to which they have experienced certain physical symptoms related to their surgery such as bruising, numbness, tingling, and swelling, using a five-point Likert scale ranging from 0 (*not at all*) to 4 (*a lot*). For the purposes of this study, we removed three items from the CROQ that refer to pain in order to avoid confusion between physical symptoms and pain. Responses were summed, ranging from 0 to 32, with higher scores indicating greater negative symptoms. Cronbach's alpha for the 8-item CROQ physical symptom subscale for this sample was 0.71.

#### Covariates: clinical, sociodemographic, and psychosocial factors

Cardiovascular history and clinical factors during admission and management were obtained from clinical notes. Clinical risk was assessed using the European System for Cardiac Operative Risk Evaluation (EuroSCORE) [21]. EuroSCORE is a combined measure of procedural mortality risk based on 17 factors comprising patient-related factors (e.g. age, sex), cardiac-related factors (e.g. unstable angina, recent MI) and surgery-related factors (e.g. surgery on thoracic aorta). Items were scored in accordance with the ‘logistic EuroSCORE’ method to generate a percentage mortality risk estimate; further details of the scoring method can be found on the EuroSCORE website (www.euroscore.org/logisticEuroSCORE.htm). In addition, the number of grafts that a participant received and whether they underwent cardiopulmonary bypass (yes/no) were also recorded. History of diabetes was taken from medical notes, categorising patients as diabetic or non-diabetic.

Participants were asked to report any longstanding illnesses apart from heart disease prior to surgery (e.g. cancer, thyroid disorder); responses were summed to compute a chronic illness burden variable. Participants were asked to specify their ethnicity by selecting from the following options: white, black or black British, mixed, Chinese, Asian or Asian British, and other ethnic group. As the majority of patients were white (88.3%) we subsequently created a binary ethnicity variable where patients were reclassified into ‘white’ and ‘non-white’ categories. Smoking was measured as a binary variable (current smoker/non-smoker). Body mass index (BMI) was assessed at the pre-operative clinic appointment and calculated using the standard formula (kg/m^2^).

Negative emotional style (NES) was assessed at baseline using a rating scale comprised of ten negative mood adjectives adapted from Cohen et al. [22]. Patients were required to rate how accurately each of the ten negative mood adjectives described how they felt during the past week using a 5-point Likert scale ranging from 1 (*I haven't felt this at all*) to 5 (*I felt this a lot*). Adjectives were derived from a factor analysis of affect items. NES comprised terms for anger (angry, hostile), depression (sad, unhappy), anxiety (tense, on edge), fatigue (tired, fatigued) and loneliness (isolated, lonely). Cronbach's alpha for the NES scale for this sample was 0.91.

### Statistical analysis

Associations between variables were examined using Pearson's correlations for continuous data and one-way ANOVAs for categorical variables. Associations between dispositional optimism, pain and physical symptom reporting were modelled using multiple linear regressions. Participants with data available for all variables of interest were included in the analyses. We included covariates that might potentially relate to pain and physical symptom reporting including ethnicity, smoking, BMI, diabetes status, chronic disease burden, cardiopulmonary bypass, number of grafts, EuroSCORE, and NES. Results are presented as both standardised and unstandardised regression coefficients and the significance level was set to *p* < .05 for all analyses, with precise *p* values reported for all test results. All statistical analyses were performed using SPSS version 20.0 (SPSS Inc., Chicago, Illinois, USA).

## Results

Table 1 summarises the characteristics of the patients at baseline, prior to CABG surgery. The sample had an age range of 44 to 90 years, was predominantly male (87.8%) and overweight (BMI > 25 = 80.7%). Approximately a quarter of patients were diabetic. The majority of patients had on-pump cardiopulmonary bypass surgery. Affective pain ratings in our sample at 6–8 weeks post-surgery ranged from 0 to 12. Pain intensity ratings ranged from 0 to 10, and ratings on the CROQ ranged from 0 to 30, indicating substantial individual differences in patient experience. Higher optimism was associated with NES at baseline (*r* = − 0.397, *p* < 0.001). Optimism was not associated with age, sex, ethnicity, BMI, chronic illness burden, management of diabetes or any other baseline factors in the analyses. Affective pain reporting was positively correlated with NES at baseline (*r* = 0.186, *p* = .009). Pain intensity was associated with BMI (*r* = 0.143, *p* = .046) and chronic disease burden (*r* = 0.159, *p* = .026). Physical symptom reporting at follow-up assessment was positively associated with NES at baseline (*r* = 0.159, *p* = .026) and number of grafts (*r* = .150, *p* = .035).

**Table 1:** Demographic, clinical and psychosocial characteristics of the sample at baseline and follow-up assessment (*N* = 197).

Characteristic	Mean ± SD or N (%)
Age (years)	68.45 ± 8.69
Female	24 (12.2)
BMI (kg/m^2^)	28.26 ± 3.77
Ethnicity — White British/Other White	174 (88.3)
Smoker	13 (6.6)

*Co-morbidities*	
Diabetes	41 (20.8)
Chronic illness burden (≥ 1)	67 (34.0)

*Clinical factors*	
Logistic EuroSCORE (%)	4.36 ± 2.97
Number of grafts	2.92 ± 1.11
On-pump	157 (79.7)

*Psychosocial factors*	
Optimism (baseline)	14.48 ± 3.35
Negative emotional style (baseline)	8.86 ± 6.44
Sensory pain (follow-up)	4.38 ± 5.41
Affective pain (follow-up)	1.13 ± 2.01
Pain intensity (follow-up)	2.34 ± 2.09
Physical symptom reporting (follow-up)	10.16 ± 5.56

### Optimism, affective pain, and pain intensity ratings at follow-up assessment

Multiple linear regressions of optimism on affective pain and pain intensity are displayed in Table 2.

**Table 2:** Multiple linear regressions of optimism on affective pain, pain intensity, and physical symptoms 6–8 weeks after CABG surgery. The bold entries indicate that these models are statistically significant (i.e. p < .05).

Model	B	SE	β	CI 95%	*p*
*Affective pain*					
Age and sex adjusted	− 0.097	0.043	− 0.162	− 0.182 to − 0.012	**.025**
Fully adjusted^a^	− 0.064	0.047	− 0.106	− 0.156–0.029	.178

*Pain intensity*					
Age and sex adjusted	− 0.111	0.045	− 0.177	− 0.199 to − 0.023	**.014**
Fully adjusted^a^	− 0.100	0.049	− 0.150	− 0.196 to − 0.004	**.042**

*Physical symptoms*					
Age and sex adjusted	− 0.368	0.117	− 0.222	− 0.598 to − 0.138	**.002**
Fully adjusted^a^	− 0.287	0.127	− 0.173	− 0.537 to − 0.036	**.025**

a Fully adjusted model: ethnicity, smoking status, BMI, diabetes status, number of grafts, number of chronic diseases, cardiopulmonary bypass, EuroSCORE, NES.

Optimism was associated with less affective pain in an age and sex-adjusted model (B = − 0.097, *p* = .025), but the relationship was no longer significant in the fully adjusted model (*p* = .18).

We found that optimism scores were negatively associated with pain intensity experienced at 6–8 weeks after CABG surgery (B = −.100, *p* = 0.042) independently of NES, and clinical and demographic factors included in the model. This suggests that patients lower in optimism experience more intense pain at 6–8 weeks post-surgery. BMI was also independently associated with pain intensity at follow-up assessment (B = 0.081, *p* = 0.046).

In order to shed more light on the nature of the relationship between optimism and post-surgical pain we decided to look at associations between pain and the optimism and pessimism subscales separately. Neither of the two subscales was significantly associated with pain outcomes, suggesting that the combined LOT score provides additional information in comparison with the two subscales.

### Optimism and physical symptom reporting at follow-up assessment

Multiple linear regressions of optimism on physical symptom reporting are displayed in Table 2.

We found a negative association between optimism and physical symptom reporting at 6–8 weeks after CABG surgery (B = − 0.287, *p* = 0.025) after adjusting for NES, clinical and demographic factors. This suggests that patients higher in optimism experience fewer physical symptoms pertaining to the CABG procedure. Number of grafts also emerged as an independent predictor of physical symptom reporting (B = 0.884, *p* = .017).

We found that the optimism subscale was not associated with physical symptom reporting (*p* = .358). However, pessimism subscale scores were significantly associated with physical symptom reporting (B = 0.470, *p* = .009) suggesting that those higher in pessimism were more likely to report more symptoms pertaining to CABG 6–8 weeks after the procedure.

## Discussion

This is the first study to our knowledge that has prospectively examined the association between optimism measured pre-surgically, and later pain reporting and elements of physical recovery specific to coronary revascularisation. The results of our study show that more optimistic patients have less intense pain up to two months after CABG surgery. Furthermore, patients higher in optimism report fewer physical symptoms pertaining to coronary revascularisation. These associations were independent of demographic and clinical factors. There was no association between optimism and affective pain reporting.

Some studies have assessed the separate contributions of the optimism and pessimism items on the LOT with health outcomes [23], [24], [25]. We therefore carried out supplementary analyses of the two subscales. The results were inconclusive, with neither the optimism subscale nor the pessimism subscale predicting later pain, though the pessimism rather than optimism subscale scores predicted later physical symptoms. These results do not provide compelling reasons for separating out the subscales in this context. The subscales are, of course, negatively correlated (*r* = −.267, *p* < .001).

Overall our findings are in line with the broader research which has reported associations between optimism and pain in several pain-related disorders [3], [4], [5], [6], [25] and post-operative pain [7], [8], [9]. Furthermore, our findings corroborate studies that have demonstrated lower physical symptom reporting in those with higher levels of optimism as measured by the LOT-R [26], [27], [28], [29], [30]. More specifically, the associations that we observed in our study are in line with previous research that has shown that a more optimistic disposition is beneficial for CABG patients in terms of rehospitalisation [14], and physical recovery and quality of life [15], [16].

Mahler and Kulik [12] previously reported associations between post-surgical optimism and pain in the weeks following CABG surgery. They also found that scores on the pessimism subscale were a more reliable predictor of post-CABG pain over time. However, Mahler and Kulik measured optimism following surgery, so it is difficult to tease apart the direction of the effect; pain may have been greater in more pessimistic individuals, but alternatively pain may have provoked a less optimistic outlook on the future.

It has been suggested that those with higher levels of optimism report less pain as they are simply less attentive to pain and better able to adjust to the pain experience [31]. Chronic pain patients higher in optimism are more likely to actively engage in diverting attention away from pain [32]. The same association has been reported in elderly patients with osteoarthritis [33]. Those with higher levels of optimism may be less likely to engage in pain catastrophising — an exaggerated negative response to pain [31]. The association between optimism and pain catastrophising has been demonstrated experimentally. Those highest in optimism engaged in less pain catastrophising prior to a painful stimulus, and those with lower pain catastrophising gave lower pain intensity ratings in response to a cold pressor test [34], [35].

Neural imaging studies have indicated that the inferior frontal gyrus, the rostral anterior cingulate, and the amygdala are involved in optimistic behaviours [36], [37]. Interestingly, each of these brain areas is involved in the processing of painful stimuli [38], [39], [40] implying cortical involvement in the association between pain and optimism. This association may also be mediated by biological markers of pain. In a recent review it was found that higher levels of pro-inflammatory cytokines such as C-reactive protein (CRP), tumour necrosis factor-α, interleukin (IL)-2, IL-6, and IL-8 were associated with greater pain in people with painful conditions [41]. Optimism has been associated with lower levels of biological markers associated with pain, such as IL-6 and CRP [42], [43] suggesting that biological markers may mediate the link between optimism and post-operative pain.

In our study we found no association between optimism and affective pain. The affective dimension of pain is more related to the emotional unpleasantness of the pain experience rather than the actual pain sensation itself [44]. Therefore, it is surprising that optimism was not associated with affective pain ratings in our study. The fact that we found associations between optimism and pain intensity in this study suggests that optimism may be more related to strength rather than quality of pain in this context. Further research in this area is required.

The strengths of this study include the prospective design, with optimism being measured approximately four weeks prior to surgery, and pain and physical symptoms being measured 6–8 weeks post-surgery. We used a well-standardised measure of optimism used in the majority of optimism studies [1] and we adjusted statistically for a number of psychosocial and clinical variables. Some authorities have argued that optimism effects are really due to confounding with negative affectivity [17]. As we included NES in our regression models, this suggests that in our study the effects of optimism are independent of negative affectivity.

There are also a number of limitations. In a meta-analysis it has been shown that the strength of the association between optimism and health is moderated by the nature of the measured outcome, in that effect sizes are stronger when health outcomes are measured subjectively [1]. This means that the use of self-report measures in our study may have inflated associations between optimism and pain and physical symptom reporting after surgery. Also, our study involved a sample largely comprised of men of white European origin meaning that the pattern of results may not be readily generalised to other groups.

In conclusion, our results indicate that a more optimistic disposition predicts less pain and fewer physical symptoms pertaining to coronary revascularisation approximately two months after CABG surgery — the time at which most patients are expected to be able to do most normal activities and return to work [45]. *Having positive expectations may promote better recovery following the CABG procedure*.

## Funding source

This study was supported by the 10.13039/501100000274British Heart Foundation (RG/10/005/28296).

## Conflict of interest

None.
